# Genetic analysis of neurodegenerative diseases

**DOI:** 10.1172/JCI199840

**Published:** 2026-04-01

**Authors:** Maurizio Grassano, Alice B. Schindler, Bryan J. Traynor, Sonja W. Scholz

**Affiliations:** 1 Neurodegenerative Diseases Research Section, National Institute of Neurological Disorders and Stroke;; 2 Neurogenetics Branch, National Institute of Neurological Disorders and Stroke; and; 3 Neuromuscular Diseases Research Section, National Institute on Aging, National Institutes of Health (NIH), Bethesda, Maryland, USA.; 4 Department of Neurology, Johns Hopkins University Medical Center, Baltimore, Maryland, USA.

## Abstract

Recent advances in genomic technologies have greatly enhanced our understanding of neurodegeneration. Techniques like whole-genome sequencing, long-read sequencing, and large-scale population studies have expanded the range of identified genetic risk factors, uncovering new disease mechanisms and biological pathways that could serve as therapeutic targets. However, translating these genetic insights into clinical practice remains difficult because of challenges in interpreting variants and the limited functional validation of new discoveries. This Review highlights the key genomic technologies advancing diagnosis and research in neurodegeneration. We focus on improvements in variant classification, detection of structural variants and repeat expansions, and combining transcriptomic, proteomic, and functional data to better determine variant pathogenicity. The ongoing integration of genomics, molecular neurobiology, and data science offers great potential for more accurate, biologically informed diagnosis and treatment of neurodegenerative disorders.

## Introduction

Genomic technologies have revolutionized the study of neurodegenerative disorders. High-throughput sequencing methods, including whole-exome sequencing (WES), whole-genome sequencing (WGS), and long-read sequencing (LRS), enabled the exploration of the human genome at an unprecedented scale (1) and at remarkably low cost (2). These tools have led to the discovery of disease-related variants (3) and broadened our understanding of how genotype relates to phenotype across a wide range of neurodegenerative syndromes, from single-gene disorders to complex, multifactorial diseases, such as Alzheimer’s disease (4), Parkinson’s disease (5), and frontotemporal lobar degeneration (6). Moreover, genetic information is increasingly shaping diagnosis, treatment decisions, and life planning (7). Nevertheless, genetic testing is still underutilized in clinical practice because of difficulties in interpreting variants and the limited clinical actionability of most late-onset risk variants.

This Review summarizes current strategies for the genetic analyses of neurodegenerative disorders, with a focus on clinical applications. We examine key sequencing technologies and how to interpret the pathogenicity of variants detected by these platforms. We also discuss integrating functional genomic data and multiomics data, such as proteomics, to enhance our understanding of the biological effects of variants. We cover the sequence of events and considerations involved in returning genomic information to patients. While examples from the neurodegeneration field are included, this Review is not meant to be an in-depth overview of genetic risk factors and disease mechanisms related to neurodegeneration.

## Sequencing technologies

**Figure 1:**
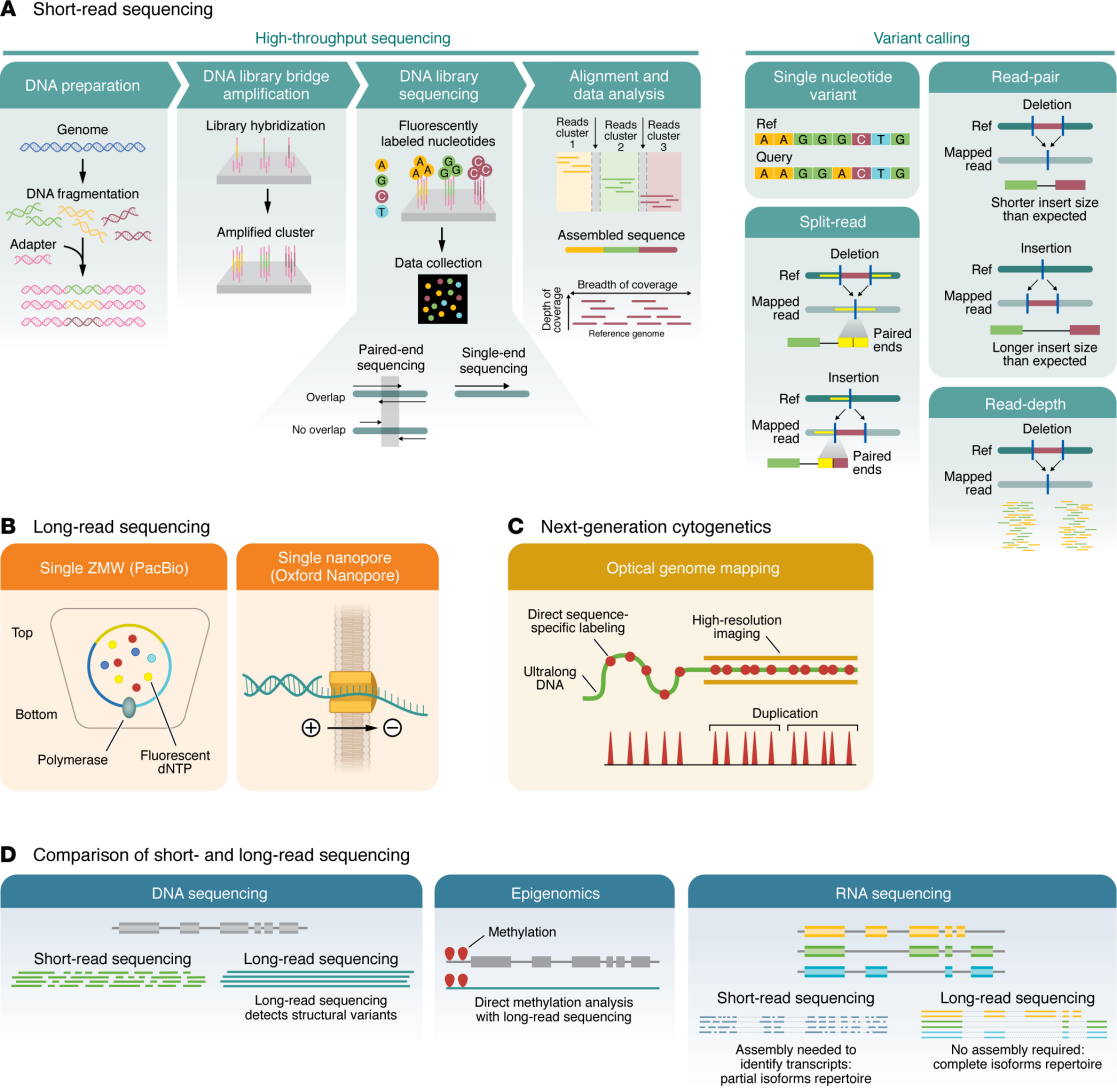
Overview of short- and long-read approaches for genetic analysis of neurodegenerative disorders. (**A**) High-throughput short-read sequencing (SRS) involves the extraction and fragmentation of genomic DNA, adapter ligation, amplification, and massively parallel sequencing, generating millions of short fragments that are aligned to a reference genome. Sequencing can be performed in *single-end mode* — where each DNA fragment is read from one end only — or *paired-end mode*, where both fragment ends are sequenced, providing orientation and insert size information that aids in detecting small structural rearrangements. Coverage depth (the number of times a given base is sequenced) and coverage breadth (the proportion of the genome covered) influence variant detection sensitivity. Variant calling algorithms for structural variants utilize distinct signatures: *read-pair signals* detect discordant spacing or orientation between paired reads, *split-read signals* identify reads that align partially to two genomic regions, and relies on local increases or decreases in coverage. (**B**) Long-read sequencing (LRS) platforms, such as Pacific Biosciences (PacBio) and Oxford Nanopore Technologies (ONT), generate reads tens to hundreds of kilobases in length, enabling more contiguous genome assembly. PacBio sequencing relies on single-molecule, real-time detection of fluorescently labeled nucleotide incorporation, while ONT senses ionic current changes as DNA or RNA molecules traverse a nanopore. ZMW, zero-mode waveguide. (**C**) Optical genome mapping provides a high-resolution, sequence-independent view of chromosomal architecture. Ultra–high molecular weight DNA is fluorescently labeled at specific sequence motifs, linearized, and imaged in nanochannels. The resulting pattern of fluorescent labels creates a physical map of the genome. (**D**) Comparative overview of sequencing technologies: LRS does not require assembly, thus enabling comprehensive DNA structural variant discovery and full-length RNA sequencing to reconstruct complete transcript isoforms. LRS also allows direct epigenetic profiling, as native DNA molecules are sequenced without chemical conversion or amplification, allowing direct detection of DNA methylation and other base modifications from characteristic signal shifts. dNTP, deoxyribonucleoside triphosphate.

Modern genetic testing for neurodegenerative disorders relies on high-throughput next-generation sequencing (Figure 1). A vital technology in this field is SRS, which supports applications from targeted gene panels to WES and WGS, each offering increasingly comprehensive genomic coverage. Unlike the older Sanger sequencing method, which reads DNA fragments one at a time, next-generation SRS platforms fragment DNA into millions of parallel reads and sequence each simultaneously (8). At the same time, advancements in computational power have allowed quick realignment and analysis of the gigabases of genomic data generated by this platform. Recently, third-generation, or long-read, sequencing technologies have emerged, generating long, continuous reads that span tens of kilobases (9).

### Targeted panels.

Gene panels are the most common first-line diagnostic tests for neurodegenerative diseases. By sequencing curated gene sets with established clinical validity, these panels provide rapid, cost-effective detection of single nucleotide variants and small indels with high precision. Their primary value is clinical actionability: identifying a pathogenic variant within a gene linked to a particular disease establishes a molecular diagnosis, guides counseling, and may inform gene-targeted therapies. The yield is highest in suspected monogenic cases with clear genotype–phenotype correlations. Expert curation of the genes included on targeted panels is critical to ensuring clinical utility (https://clinicalgenome.org/) (10).

Interpretation of variants requires integration of variant- and gene-level evidence. The “ABC” framework distinguishes the likelihood that a variant impacts gene function (A) from the strength of gene–phenotype validity (B), enhanced with standard comments (C) (11). This strategy complements the American College of Medical Genetics–based (ACMG-based) classification (12), the international standard, and reduces misclassification in complex cases (13). Automated tools support the standardized application of ACMG guidelines and reduce interrater variability (14, 15), though expert review remains indispensable, especially in neurodegenerative disorders, where incomplete and age-dependent penetrance complicates counseling. Moreover, variants of unknown significance (VUS) classifications disproportionately affect individuals from non-European ancestries (16). Nonetheless, targeted panels remain the most practical entry point for clinical genetic testing in neurodegeneration, especially for disorders with known genetic architectures.

### Whole-exome and whole-genome sequencing.

Whole-exome and whole-genome sequencing (WES and WGS) are replacing targeted panels because of technological advances, lower costs, and their more comprehensive coverage. WES focuses on coding and splice site regions, capturing most known pathogenic variants (17, 18), but has inconsistent exon coverage and provides incomplete coverage of noncoding regions. WGS overcomes this limitation by providing uniform, unbiased coverage across nearly the entire genome. In addition, WGS improves detection in complex regions and can identify structural and repeat variants more effectively than karyotyping, microarrays, or multiplex ligation-dependent probe amplification (19, 20). Genome sequencing has a broad scope and long shelf life: existing WGS data can be reanalyzed as new genes are discovered, without the need for repeat sequencing. This shift has moved the bottleneck in genetic testing from variant detection to variant interpretation — specifically, distinguishing the few clinically meaningful variants from millions of benign ones (21).

### LRS.

LRS analyzes long DNA strands, providing a more comprehensive view of the genome than short-read methods (22). Two platforms lead the field: PacBio’s Single Molecule Real-Time monitors DNA synthesis in real-time by sequencing circular templates; Oxford Nanopore infers DNA sequences from electrical current disruptions as DNA molecules pass through nanopores. Both are rapidly improving but involve trade-offs in accuracy, cost, and throughput (22). The main advantage of LRS is its ability to resolve complex genomic features that short-read technologies cannot assess. For instance, recent assemblies using LRS have added over 200 million base pairs to the human reference genome, resolving repetitive and structurally complex regions that may contain variants linked to neurodegeneration (1). Additionally, by spanning entire repeat expansion regions, LRS can accurately measure repeat lengths, detect sequence interruptions, and reveal instability patterns (23). LRS applications go beyond DNA sequencing. Full-length RNA transcript sequencing reveals alternative isoforms that short-read RNA sequencing often misses. In addition, direct detection of DNA methylation with Oxford Nanopore allows the identification of imprinting defects and broader epigenetic dysregulation associated with neurological diseases (24).

LRS is poised to revolutionize neurogenetics and is increasingly used alongside SRS. Still, barriers like high costs, strict DNA input needs, higher error rates, less advanced bioinformatics pipelines, and less comprehensive reference databases hinder broader adoption, complicating the interpretation of complex variants.

## Variant interpretation

**Figure 2:**
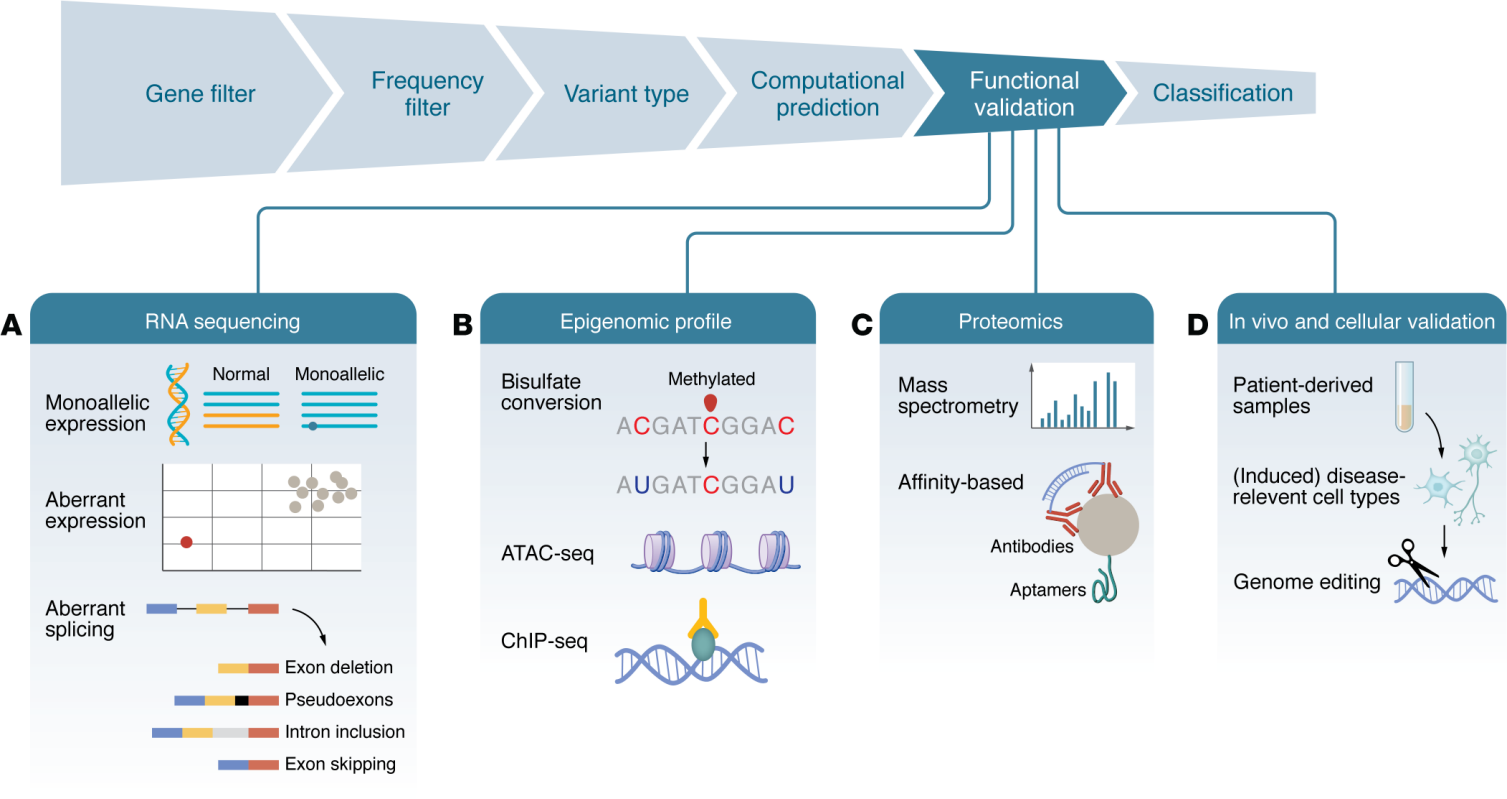
Integrative variant filtering and functional validation strategies. After variant detection, successive filtering steps are applied to prioritize candidate variants. Functional validation integrates multiomics and experimental approaches to assess the biological impact of prioritized variants. (**A**) Transcriptomic validation by RNA sequencing (RNA-seq) can reveal molecular consequences of candidate variants at the transcript level. Abnormal expression of the affected gene may indicate altered transcriptional regulation or mRNA stability. Monoallelic expression suggests allelic imbalance, often due to nonsense-mediated decay or promoter methylation. Aberrant splicing may include exon skipping (canonical exons omitted), intron retention (intronic sequences retained due to splice site disruption), pseudoexon inclusion (cryptic intronic sequences aberrantly incorporated), or shortened or extended exons, resulting from alternative splice donor or acceptor usage. (**B**) Epigenomic methods assess DNA and chromatin modifications that influence gene expression. Bisulfite sequencing provides base-resolution maps of cytosine methylation. Assay for transposase-accessible chromatin sequencing (ATAC-seq) identifies regions of open chromatin, marking regulatory regions, such as promoters and enhancers. Chromatin immunoprecipitation sequencing (ChIP-seq) maps histone modifications or transcription factor binding. (**C**) Proteomic validation: Proteomic analyses evaluate the downstream impact of variants on protein abundance and function. Mass spectrometry can be used in qualitative modes to identify protein isoforms or posttranslational modifications and in quantitative modes to measure differential protein levels across samples. Complementary affinity-based platforms, such as Olink or SomaScan, provide targeted, high-throughput quantification using specific molecular binders. (**D**) In vivo and cellular validation: Functional assessment of variant pathogenicity often employs patient-derived samples, such as fibroblasts or induced pluripotent stem cells, which can be differentiated into disease-relevant cell types (e.g., neurons). CRISPR-based genome editing enables introduction or correction of variants to test causality, and rescue experiments — where reexpression of the wild-type allele restores normal function — provide strong evidence of pathogenicity.

With this understanding of the available sequencing technologies, we now turn to variant interpretation (Figure 2 and Table 1 and Table 2). A simple approach is to report only confirmed pathogenic variants to the physician and patient. However, this may overlook a sea of complexity within which lies the potential to identify pathogenic variants and to improve diagnostic yields. Several strategies for interpreting variants exist, but none are perfect on their own. The field, therefore, emphasizes combining multiple data sources to assess pathogenicity.

### Population frequency filtering.

The first step in variant prioritization typically involves filtering out common polymorphisms by consulting large multiancestry population reference databases, like the Genome Aggregation Database (gnomAD; https://gnomad.broadinstitute.org/) (25) and Trans-Omics for Precision Medicine (TOPMed; https://topmed.nhlbi.nih.gov/) (26). For dominantly inherited disorders, variants with a minor allele frequency greater than 0.1% are usually excluded, while more lenient thresholds may be applied for recessive diseases (27). However, careful interpretation is crucial, as variants rare in one ancestry may be common in another. Reference datasets may also include carriers of risk alleles for late-onset neurodegenerative diseases (17). Furthermore, despite recent effort to improve the diversity of samples, non-European ancestries remain underrepresented in population datasets (16).

### Predicting pathogenicity.

Loss-of-function alleles, such as stop codons, frameshift indels, or canonical splice site variants, in genes already linked to neurodegenerative disease are generally considered pathogenic. However, healthy individuals can also carry loss-of-function alleles (28), which are typically benign. The pathogenicity of missense variants is more difficult to interpret. Early prediction tools relied on evolutionary conservation (29, 30) and protein structure data (31). Over time, many algorithms were developed (32), resulting in a crowded field without a clear consensus on the most reliable methods. Modern ensemble models improve performance by combining multiple algorithms (33–36) (Table 1). Recently, machine learning models, such as AlphaMissense (37) and ESM1b (38), trained on protein sequence, have shown promising results, though their widespread clinical utility remains untested (39). Benchmarking efforts, such as Critical Assessment of Genome Interpretation (40), highlight the strengths and limitations of these models, which are influenced by biases in training data and inaccuracies in reference annotations. Importantly, prediction scores alone do not confirm disease causality and should always be considered alongside other evidence.

### Structural variants.

Structural variants are genomic rearrangements larger than 50 base pairs, including deletions, duplications, insertions, inversions, translocations, and complex rearrangements. Structural variants play a crucial role in neurodevelopmental disorders (41, 42) and contribute to neurodegeneration (43). For instance, *SNCA* and *APP* copy number gains cause autosomal dominant Parkinson’s disease (44) and Alzheimer’s disease (45), respectively, via dosage effects, and the *MAPT* inversion characterizes major haplotypes associated with tauopathy (46). Genome-wide studies have identified pathogenic structural variants in known Alzheimer’s genes (*SORL1*, *ABCA7*, *APP*) (47), as well as variants in Lewy body dementia, ALS, and FTD (48). Including structural variant detection in WGS pipelines can greatly improve diagnostic yield and expand understanding of pathogenic mechanisms.

Detecting structural variants in short-read WGS data combines different signals using multialgorithm pipelines to enhance detection (49). As this approach is often considered cumbersome and imprecise, LRS is gaining acceptance for clinical use. Information on whether structural variants overlap coding regions or other known protein-altering DNA segments can help assess their pathogenicity. Conversely, interpreting structural variants that do not directly impact coding regions remains challenging. Nonetheless, population catalogs (50–53) integrating genomic and functional data are emerging to support this effort.

### Repeat expansions.

Short tandem repeat expansions (STRs) are a key cause of neurodegeneration (54). Classic examples include the [CAG]n expansion in *HTT* in Huntington’s disease (55) and the [GGGGCC]n hexanucleotide repeat expansion in *C9orf72*, which is the leading genetic cause of ALS and FTD (56). Traditional repeat expansion detection relied on targeted assays, such as repeat-primed PCR or Southern blotting, which are labor-intensive, locus specific, and unsuitable for broad diagnostic screening. WGS, combined with specialized computational tools such as ExpansionHunter (57, 58), can now estimate a repeat size even when expansions exceed the read length, transforming STR detection. These methods achieve remarkably high sensitivity and specificity (>97% and >99%, respectively) (59) across various loci and can be applied directly to existing short-read WGS datasets, enabling simultaneous assessment of multiple loci. Although PCR confirmation remains necessary for novel loci, the high precision of WGS-based repeat expansion detection, supported by large population-scale STR reference databases (60), reduces the need for additional laboratory-based validation.

Perhaps most notably, population-scale WGS has revealed the widespread presence and variability of STRs. Recent studies indicate that pathogenic-range expansions are more common than thought (61, 62), affecting about 1 in 283 people (59). Many carriers show no symptoms, which has clinical implications, supporting broader testing and suspicion in individuals with compatible phenotypes and requiring careful counseling of asymptomatic individuals (61).

### Noncoding variants.

A major hurdle in genome sequencing is assessing the pathogenicity of rare variants in noncoding regions. Initially, diagnostic efforts targeted only canonical splice sites. However, recent evidence indicates that pathogenic variants also occur in deep intronic regions, cryptic splice sites, and exonic splicing enhancers, often with effects as severe as protein-coding mutations (63). Advanced deep learning algorithms predict the disruptive effects of variants on splicing (64, 65), identifying mutations that may create cryptic or pseudoexons and that previously went undetected. In addition to splicing, WGS enables the detection of variants in regulatory elements, such as promoters, untranslated regions, enhancers, and noncoding RNA genes. Nevertheless, determining the pathogenicity of rare variants in these regions remains especially challenging in neurodegeneration because of the brain’s extensive alternative splicing, where even minor regulatory disturbances can have substantial effects. Emerging methods, such as intronic constraint metrics and the integration of chromatin accessibility and tissue-specific expression data, provide probabilistic insights into the impacts of noncoding variants but are primarily used in research contexts (66). Functional studies in disease-relevant cells — such as expression analysis, reporter assays, or CRISPR experiments — are essential for confirming noncoding variant effects.

### mtDNA.

WGS can offer reliable mitochondrial genome coverage, but its precise evaluation requires tailored pipelines that align circular DNA, account for nuclear homologs, and identify heteroplasmy. Pathogenic mtDNA variants have been observed in patients with neurological disorders (67), but they can also attenuate neurodegeneration (68). Since mtDNA variation increases with age and might indicate cell-specific dysregulation (69), analyzing neurons derived from accessible patient tissues can provide more relevant insights for disease interpretation.

### Family-based studies.

Family data are crucial for validating candidate variants. In early-onset disorders, parent–offspring trios quickly identify de novo variants, reduce the number of candidate variants, and help in phasing compound heterozygotes. However, trio studies are rarely feasible in late-onset neurodegeneration because the parents are unavailable. Although de novo mutations in genes like *PRKN*, *APP*, *SOD1*, and *FUS* have been documented (70–72), their overall significance remains uncertain (73, 74). Segregation analysis is valuable in multigenerational pedigrees but must consider age-related penetrance, variable expressivity, and phenocopies, which can obscure or falsely suggest cosegregation. LRS can enhance segregation studies by determining haplotype phase and parental origin. Finally, family studies can help in analyzing cases with multiple rare variants, as well as in assessing modifier effects and oligogenic risk.

### Functional validation.

Assessing the pathogenicity of new or uncertain significance variants requires some level of functional validation. Functional assays directly measure how variants affect gene expression, protein activity, or cellular pathways. Traditionally, these tests were conducted individually for each variant. However, the advent of multiplexed assays of variant effect (MAVEs) has enabled high-throughput testing of thousands of variants simultaneously. This is done by introducing large libraries of variants into model systems and analyzing the outcomes through sequencing-based readouts (75). Deep mutational scanning assesses how coding variants affect protein activity or stability, while massively parallel reporter assays examine the regulatory roles of noncoding variants. Together, these methods can create detailed sequence-function maps.

The clinical usefulness of MAVE data is progressing. Frameworks now incorporate MAVE data into ACMG classification (76, 77), and resources like MaveDB (https://www.mavedb.org) support gene-specific interpretation (78). Since MAVE evidence is independent of frequency, it is especially valuable for underrepresented populations, where VUS rates remain high (79). However, results depend heavily on assay design (75) and must be tailored to accurately reflect human neuronal biology and neurodegenerative mechanisms, as shown by MAVE-inspired models adapted for variants in *LRRK2* (80) and *APP* (81). Definitive validation typically involves additional experiments using model organisms or patient-derived cells, such as employing CRISPR to introduce patient variants or creating isogenic controls to evaluate disease-related impacts on neuronal function and survival (82).

### RNA-seq.

RNA-seq analyzes gene expression, transcript integrity, and splicing to enhance variant classification and uncover disease mechanisms that DNA tests might miss (83). It increases diagnostic success in Mendelian disorders by 7% to over 30% by detecting abnormal RNA patterns, such as unusual expression, monoallelic expression, and splicing defects (84–89). Automated computational tools aid in identifying these phenomena (90–93). This is especially important in the brain, where alternative splicing plays a key role in defining neuronal identity (94) and is markedly altered in neurodegenerative diseases (95), as illustrated by the widespread cryptic exon inclusions in ALS caused by TDP-43 dysfunction (96).

### Multiomics.

Proteomics allows observation of genetic variants’ downstream effects by measuring protein levels and detecting unusual expression patterns. Consequently, proteomic data refine the interpretation of variants linked to genomic or transcriptomic findings (97). Coanalyzing RNA expression, protein levels, and other omics, such as DNA methylation, has resulted in small but meaningful improvements in diagnosis (97, 98). For example, proteomics can highlight pathogenic variants in mitochondrial disorders when DNA findings are inconclusive (97). Similarly, integrated transcriptomic and proteomic analyses in patient-derived cells resolve unsolved cases by uncovering splicing defects and protein dysregulation (98). Tissue context is crucial. Brain tissue is rarely accessible, but skin fibroblasts or blood cells can be alternatives. Neuronal cells can be derived from these via standard induced pluripotent stem cell protocols, which, though costly and time-consuming, enable studying neuronal transcripts and proteins. Notably, differentiation of induced pluripotent stem cells typically yields developmentally immature neurons. Direct transdifferentiation approaches (99), which convert somatic cells into neurons without a pluripotent intermediate, may better preserve age- and disease-associated cellular and epigenetic features.

## Genetic testing: clinical setting

Integrating WGS into clinical practice requires balancing large genomic datasets with interpretation challenges. For neurological disorders, treating WGS as a versatile test within a structured, step-by-step diagnostic process improves outcomes and manages complexity (Table 2). Genetic counseling links the laboratory, neurologist, geneticist, and family, and it must be adaptable.

### Genetic counseling.

Consensus guidelines published by the ACMG and the AMP set standards and offer structured frameworks for classifying variants and informing patients (100). Interpreting these variants in neurodegenerative diseases is complicated by variable expressivity and incomplete penetrance (101), making counseling particularly difficult. Family-based estimates of penetrance often overstate risk because they come from cohorts with many affected individuals, whereas biobanks may underestimate risk because of healthier participants and limited phenotyping. Family history remains the most trusted risk assessment method. Evidence shows the impact of rare pathogenic variants largely depends on whether close relatives are affected (102). Consequently, a variant with moderate penetrance can imply very different risks in familial versus sporadic cases. Machine learning models are being developed to improve quantitative risk estimates (103). However, until disease-specific models become accessible, risk assessment should combine variant data with family history to prevent overdependence on population estimates.

### Genetic testing.

The decision of when to perform genetic testing is primarily guided by the diagnostic yield and clinical significance. On one hand, testing is crucial for patients with early-onset or familial diseases, such as many ataxias, choreiform disorders, and hereditary spastic paraplegias, where molecular confirmation resolves diagnostic uncertainty. Recently, the development of gene-targeted therapies has created new impetus for diagnostic testing. For instance, the availability of *SOD1*-targeted therapy has made testing a necessity in ALS cases (104). Similar trends are seen in Alzheimer’s disease, where *APOE ε4* genotyping guides decisions about antiamyloid therapies (105). Overall, these advances mark a shift to genetically informed neurodegeneration care, where genetic results aid diagnosis and guide therapy. As precision treatments evolve, genetic counseling and testing will become increasingly vital components of neurological care. Importantly, even in the absence of direct therapeutic actionability, genetic testing may offer benefits that meaningfully influence patient decision-making, including improved diagnostic clarity, psychological well-being, informed life and family planning, cascade screening of relatives, access to support resources, and contribution to ongoing research (106, 107). Ultimately, genetic testing requires a multidimensional patient-centered approach that balances emerging but limited therapeutic opportunities with broader clinical and personal considerations (Table 3).

### Stepwise analysis.

Clinical genetic testing typically starts with short-read WGS, followed by virtual panels for relevant genes based on the clinical phenotype. If these are inconclusive, the same data can be reanalyzed later without resequencing. LRS is often used next, especially for structural variants or pathogenic repeat expansions. Method choice depends on cost and disease complexity; surprisingly, sequential testing can be more expensive and less efficient than direct WGS, especially in complex neurodegenerative diseases with multiple variant types. For conditions with known genetic causes, targeted panels are still a practical first step.

### Reanalysis.

WGS data can be reinterpreted over time as genomic knowledge advances, enabling new diagnoses months or years later. This iterative, ongoing approach is crucial in neurodegenerative diseases, where understanding is incomplete but evolving, making WGS a long-term diagnostic resource. Systematic sequencing enables population-scale discoveries, deepening disease understanding and broadening genomic testing benefits beyond individuals. However, realizing this potential requires secure long-term data storage and coordinated systems for reinterpreting variants.

### Incidental and secondary findings.

The broad scope of WGS often uncovers findings beyond the primary clinical indication. Incidental findings may emerge unintentionally, while secondary findings are intentionally sought in curated lists of actionable genes. Guidelines vary on how to handle these (108). The ACMG recommends routinely reporting actionable variants for prevention and early intervention (109). In contrast, the European Society of Human Genetics prefers a narrower approach to unsolicited information (110). Thorough pretest counseling, explicit informed consent, and structured disclosures are essential to help patients understand the potential impact of secondary findings on their health and family decisions.

## Genetic testing: research setting

**Figure 3:**
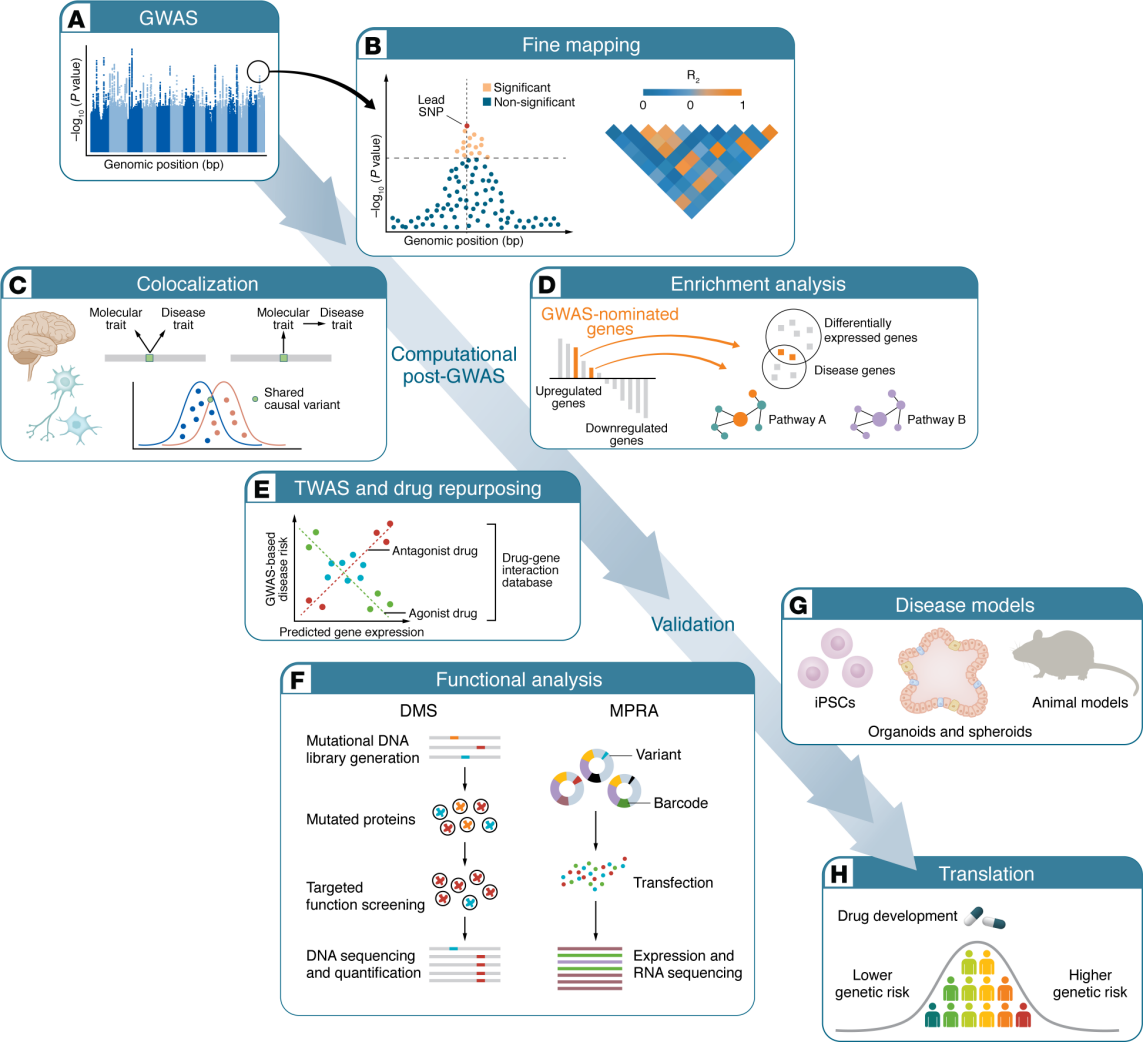
From genome-wide association to functional and translational insights. (**A**) Genome-wide association studies (GWAS) identify common genetic variants associated with phenotypes. Significant associations, visualized as peaks in a Manhattan plot, highlight genomic loci linked to disease susceptibility but often encompass multiple correlated variants within linkage disequilibrium (LD) blocks. (**B**) Fine mapping integrates LD structure and effect sizes to calculate the posterior probability that each variant is causal. Conditional analysis iteratively accounts for the most important variants within a locus to identify independent association signals. (**C**) Colocalization analysis tests whether GWAS and molecular quantitative trait locus (QTL) signals share a common causal variant. Expression QTL (eQTL) mapping associates genetic variants with gene expression levels across tissues or cell types, helping prioritize effector genes and relevant biological contexts. (**D**) Aggregating signals across genes or pathways enables the identification of biological processes disproportionately affected by disease-associated variants. Gene set enrichment, tissue-specific expression profiling, and network-based methods reveal convergent mechanisms. (**E**) Transcriptome-wide association studies (TWAS) integrate GWAS summary statistics with reference transcriptomic data to infer associations between genetically predicted gene expression and disease. Integration with drug–gene interaction databases facilitates drug repurposing by identifying compounds that counteract disease-associated expression profiles or target implicated pathways. (**F**) High-throughput reporter-based assays, such as massively parallel reporter assays (MPRAs), test the regulatory activity of thousands of variants simultaneously, while deep mutational scanning (DMS) assesses the functional impact of coding variants on protein stability or activity. (**G**) Cellular and organismal models provide experimental validation of causal mechanisms. Patient-derived cells, patient-derived organoids, and genetically engineered animal models enable functional interrogation of candidate variants and pathways in disease-relevant systems. (**H**) Insights from GWAS and post-GWAS analyses inform therapeutic development and precision medicine. Polygenic risk scores integrate the cumulative effects of multiple variants to stratify disease risk and predict progression, while genetic insights guide drug discovery and repurposing efforts targeting causal genes or pathways.

Here, we discuss how the genetic architecture of neurodegenerative diseases is being studied in research labs (Figure 3). The information is usually not immediately applicable to clinical practice, but it lays the groundwork for future clinical use. Therefore, it is essential to explain how these research procedures work so that clinicians can assess the reliability of data supporting their future clinical decisions.

### GWAS.

The unmatched workhorse for uncovering the genetic basis of neurodegenerative diseases is the GWAS. By comparing millions of genetic variants between affected and unaffected individuals in large cohorts (111), GWAS revealed that most age-related neurodegenerative disorders stem from the cumulative effect of many common variants. While each variant individually confers only a small increase in risk (4, 112–117), collectively they account for a large portion of heritability.

GWAS offers a unique view of neurodegenerative diseases but has limitations. Linkage disequilibrium causes GWAS signals to highlight large genomic regions instead of specific genes or variants. Many associations are in noncoding regulatory elements, implying risk stems from regulatory changes. Interpreting GWAS results, therefore, involves mapping signals to causal variants, effector genes, and pathways (118, 119). Proximity-based annotations can be misleading, as regulatory effects may span large genomic distances and are cell type specific. Incorporating functional genomics allows for variant- and gene-focused interpretation within network-level models of gene regulation (120–122). The following sections explore how geneticists connect genetic associations to robust biological data and, ultimately, actionable clinical insights.

### Quantitative trait loci.

A molecular quantitative trait locus (QTL) is a specific genetic site where variation influences a measurable cellular trait. The most common trait studied is expression, where an allele correlates with changes in the level of a particular transcript; these are called expression QTLs (eQTLs). Other trait loci include alternative splicing, protein levels, and DNA methylation, among others. When a GWAS signal overlaps with a molecular QTL in a relevant tissue, it strongly suggests the mechanism by which the GWAS signal affects the cell and contributes to disease (123). This often also points to a potential causal gene in the region. Colocalization analysis, combining GWAS and molecular QTLs, is key for understanding association signals. However, genetic variants influence molecular traits differently across tissues and cell types, and these biological contexts must be taken into account (124, 125).

### Transcriptome- and proteome-wide association studies.

Transcriptome- and proteome-wide association studies (126, 127) utilize QTL reference panels to link genetically predicted gene expression or protein abundance to disease risk. Essentially, TWAS combines eQTL data with GWAS effect sizes to assess whether predicted gene expression is associated with disease susceptibility. This approach often identifies potential effector genes even when the causal variants are unclear. TWAS also allows for estimating neuronal and glial expression at disease onset without requiring brain biopsies (128). These frameworks have also been used to demonstrate that many genetic risk factors involved operate outside the central nervous system, for example, implicating monocytes in Parkinson’s disease (129).

### Genetic pleiotropy and causal analysis.

Making GWAS results publicly accessible, whether as individual genome data or summary statistics, has been vital for advancing large-scale genetic research. Pooling summary data across multiple studies in meta-analyses enhances the detection of genetic signals and lowers false-positive rates (130). Cross-trait genetic correlations have expanded our knowledge of pleiotropy in neurodegeneration, uncovering overlaps among Alzheimer’s disease, Parkinson’s disease, and the ALS-FTD spectrum (131). Mendelian randomization helps identify causal links between diseases and traits (132), including modifiable factors (133, 134). In neurodegeneration, it has revealed the involvement of lipid metabolism (135–137) and circulating inflammation biomarkers (138–140) and highlights potential risk proteins (141). Most importantly, using summary data speeds up drug development by enabling systematic colocalization of disease loci with drug targets (142, 143) or simulating drug effects via Mendelian randomization (135, 144, 145). Robust conclusions, however, depend on thorough sensitivity analyses and transparent reporting (146, 147).

### Limitations of GWAS.

Despite progress, GWAS approaches still face several limitations. Most GWAS focus on European-ancestry cohorts, limiting the generalizability of the results and impeding the discovery of new risk loci. Progress is also hampered by restrictions on sharing DNA samples and GWAS summary statistics. Furthermore, the ability of GWAS to detect an association signal of low effect depends on sample size and the accuracy of the clinical phenotyping of the included samples. In this regard, the recent trend of using proxy phenotypes, such as family history or electronic health record codes, instead of neuropathologically or biomarker-confirmed diagnoses, is concerning because it introduces heterogeneity and misclassification (148). Emerging designs address these issues by focusing on quantitative endophenotypes more closely linked to disease pathology. For example, GWAS of cerebrospinal fluid biomarkers identified loci associated with amyloid processing or tau phosphorylation (149, 150), revealing previously unrecognized mechanistic subtypes in Alzheimer’s disease. Similar strategies utilizing neuropathology (151); neuroimaging (152, 153); or clinical features, such as disease-specific age at onset (154) or progression measures (155), can reduce heterogeneity, enhance biological interpretability, and shed light on early pathogenic processes.

### Polygenic risk.

Individual GWAS loci have limited predictive value and are of minimal use in clinical settings. PRS address this issue by capturing the genetic complexity of neurodegenerative diseases, where many variants interact to influence risk. Essentially, a PRS sums the effects of these variants to estimate an individual’s inherited disease risk (156, 157). This approach has proven effective in neurodegeneration: individuals in the highest PRS percentiles for Alzheimer’s disease face several times greater odds of developing the disease than those at median levels (158–160). Currently, PRS is mainly used in research to stratify individuals enrolled in biomarker studies, clinical trials, and observational cohorts. Recent studies suggest that adjusting for polygenic backgrounds also improves the analysis of rare variants (161). In neurodegeneration, disease-specific PRS enable prediction of the effect of highly penetrant *LRRK2* mutations (162) and explain differences in age of onset among *APOE ε4* allele (163) and *C9orf72* expansion carriers (143). However, clinical use remains limited because PRS’s predictive power is still modest, constrained by incomplete knowledge of disease genetics and the exclusion of environmental factors (164, 165). Additionally, the transferability of PRS across diverse populations is limited by variations in allele frequencies and linkage disequilibrium, despite some recent progress (166, 167). As PRS will undoubtedly be increasingly used in clinical settings, it will be paramount to provide careful counseling and to clearly communicate the uncertainties associated with these scores (165, 168, 169).

### Novel approaches.

Bulk RNA-seq may obscure cell type effects in brain disorders, where neurons, glia, and vascular cells differ in function and vulnerability. Techniques like single-cell and single-nucleus sequencing reveal cell type–specific variants, vulnerabilities, and disease-related cellular states, including responses to tau in neurofibrillary tangles in Alzheimer’s disease (170). Spatial transcriptomics is an emerging technology that preserves tissue architecture while mapping gene expression, enabling precise visualization of gene activity at the cellular level across the brain (171). Analyses of spatial transcriptomes have indicated that immune activation and microglial upregulation occur before motor neuron loss in ALS (172). In Alzheimer’s disease, proximity-based studies of amyloid plaques have shown that involvement of myelin and oligodendrocytes happens earlier than previously believed. Additionally, a multicellular pattern with complement activity, inflammation, and oxidative stress was detected mainly in later stages (173, 174). There is growing hope that this information can enhance our understanding of how patient variants from WGS impact biology, especially if large datasets become publicly accessible.

### Artificial intelligence.

Artificial intelligence (AI) is becoming increasingly vital for translating genomic information into results that can be used clinically (175). Specifically, machine learning techniques improve variant analysis and genotype–phenotype correlation, while natural language processing accelerates curation by extracting important clinical and literature details (Tables 4 and 5) (176). Large language models have the potential to enhance communication before and after testing and might even assist in identifying patients who could benefit from genetic evaluation (176, 177). In the future, incorporating AI into current systems is expected to reduce uncertainty and promote equity and transparency, though human oversight will continue to be necessary for the foreseeable future.

## Conclusion

The increased availability of WGS and LRS is transforming genetic testing from research into an essential component of clinical evaluation. As genomic testing becomes more common, interpretation must use structured frameworks that incorporate variant data, phenotype information, family history, and molecular plausibility. This shift necessitates thorough pre- and posttest counseling to manage uncertain findings, incomplete penetrance, and secondary results, thereby ensuring genomic data support clinical decisions. Additionally, the gap between research and clinical practice is closing, driven by advances in genomics and multiomics technologies. These innovations are enhancing our understanding of neurodegeneration. Continued progress relies on collaboration among neurologists, geneticists, data scientists, and molecular biologists, working together to interpret genomic data within a biological framework. Ultimately, such interdisciplinary efforts will foster a more biologically based approach to diagnosing neurodegenerative diseases and usher in the era of precision medicine.

## Author contributions

MG and SWS conceptualized the outline of the review article. MG wrote the initial draft of the manuscript and designed display items. ABS, BJT, and SWS critically reviewed and edited the manuscript.

## Funding support

This work is the result of NIH funding, in whole or in part, and is subject to the NIH Public Access Policy. Through acceptance of this federal funding, the NIH has been given a right to make the work publicly available in PubMed Central.

- Intramural Research Program of the NIH (program: ZIANS003154, ZIAAG000933).

## Figures and Tables

**Table 3:**
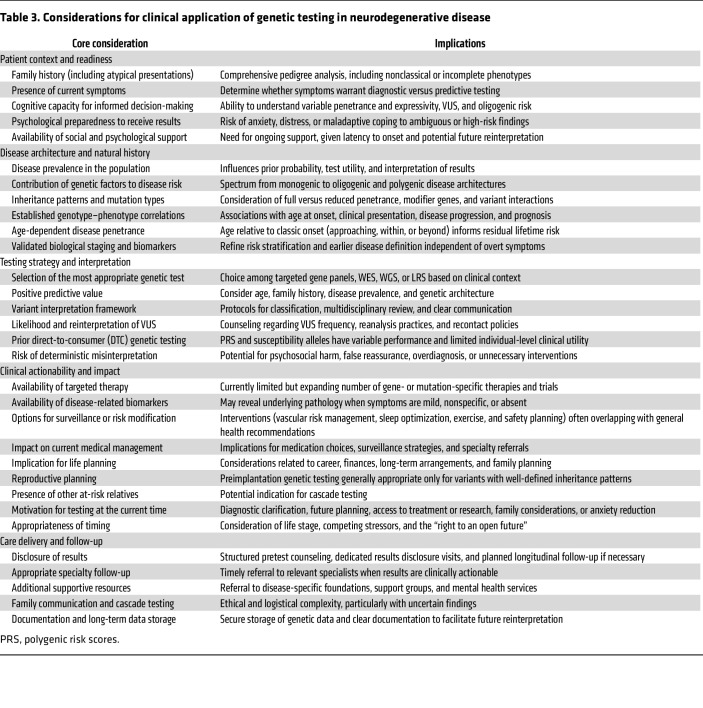
Considerations for clinical application of genetic testing in neurodegenerative disease

**Table 1:**
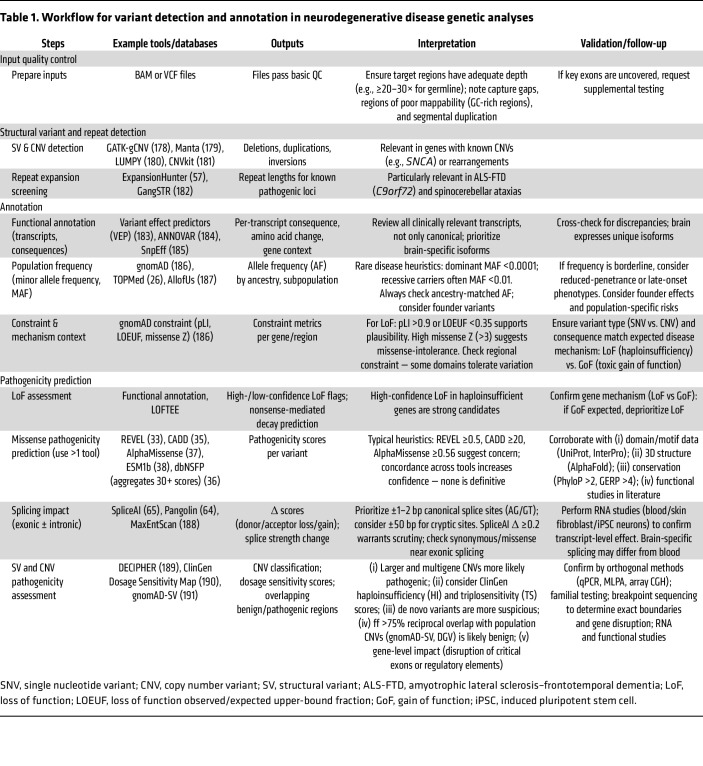
Workflow for variant detection and annotation in neurodegenerative disease genetic analyses

**Table 2:**
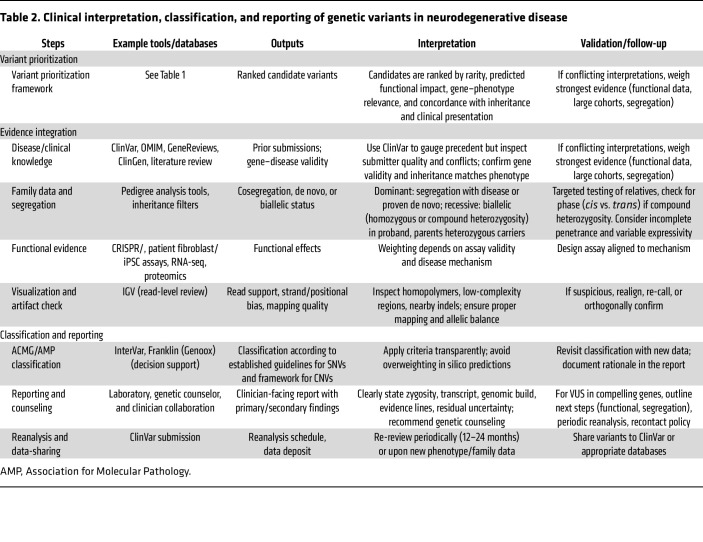
Clinical interpretation, classification, and reporting of genetic variants in neurodegenerative disease

**Table 4:**
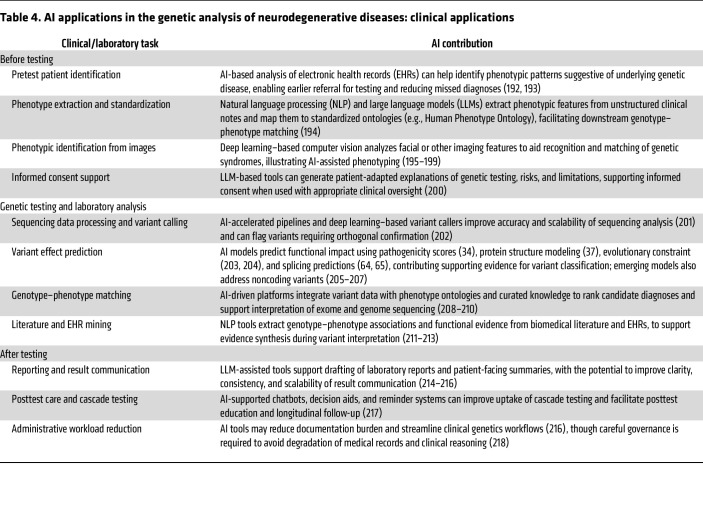
AI applications in the genetic analysis of neurodegenerative diseases: clinical applications

**Table 5:**
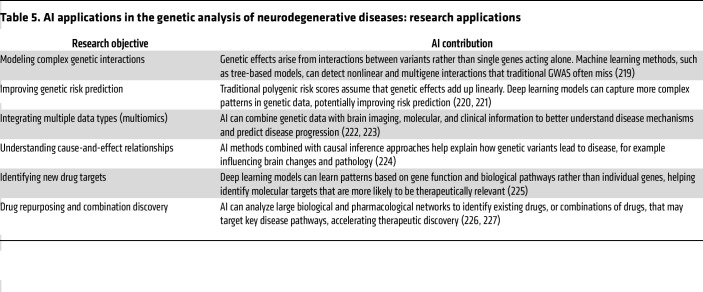
AI applications in the genetic analysis of neurodegenerative diseases: research applications

## Version 1 04/01/2026

Electronic publication
